# Looking at individual symptoms: the dynamic network structure of depressive symptoms in cancer survivors and their preferences for psychological care

**DOI:** 10.1007/s11764-022-01246-4

**Published:** 2022-08-17

**Authors:** E. A. Bickel, M. P. J. Schellekens, J. G. Smink, V. E. M. Mul, A. V. Ranchor, J. Fleer, M. J. Schroevers

**Affiliations:** 1 Department of Health Psychology, University of Groningen, University Medical Center Groningen, Groningen, The Netherlands; 2 Centre for Psycho-Oncology, Scientific Research Department, Helen Dowling Institute, De Bilt, The Netherlands; 3 Tilburg School of Social and Behavioral Sciences, Department of Medical and Clinical Psychology, Tilburg University, Tilburg, The Netherlands; 4 Department of Radiation Oncology, University Medical Center Groningen, Groningen, The Netherlands

**Keywords:** Psychological care needs, Depressive symptoms, Cancer, Network approach

## Abstract

**Purpose:**

The majority of depressed cancer survivors do not receive psychological care, possibly because offered care does not align with their experiences and preferences. We examined (1) which depressive symptoms cancer survivors would like to receive psychological care for; (2) how distinct depressive symptoms are related to each other in the contemporaneous and temporal network of depressive symptoms; and (3) whether survivors’ care needs correspond to the interconnectedness of these specific symptoms.

**Method:**

Fifty-two cancer survivors suffering from at least mild depressive symptoms and were not receiving psychological care filled out a baseline questionnaire about their care needs for distinct depressive symptoms, followed by ecological momentary assessments (EMA) assessing depressive symptoms (14 days, five times a day). Multi-level vector autoregression analysis was used to estimate associations between distinct depressive symptoms as well as their centrality within the network.

**Results:**

Cancer survivors most strongly preferred to receive care for fatigue, feeling down, little enjoyment, and sleep problems. Fatigue, together with worry and lack of concentration, most strongly predicted the onset of other symptoms. Little enjoyment and feeling down were two of the most central symptoms (i.e., strongly connected to other symptoms) in the contemporaneous network and were most strongly influenced by other symptoms in the temporal network.

**Conclusions:**

Clinicians can offer specific interventions that target fatigue, as these played an important role in the onset of symptoms and would align with survivors’ needs.

**Implications for Cancer Survivors:**

Offering such symptom-specific care may increase the uptake of psychological interventions in cancer survivors.

**Supplementary Information:**

The online version contains supplementary material available at 10.1007/s11764-022-01246-4.

## Introduction

Up to a quarter of people diagnosed with cancer suffer from depressive symptoms [1, 2]. This is higher than in the general population [3] and these symptoms can negatively impact their quality of life [4, 5]. Effective evidence-based treatments for reducing depressive symptoms are available, but only a quarter of people with cancer and depressive symptoms accept and receive such treatment [6, 7]. One reason for this low care uptake might be that the offered care for depressive symptoms does not match with the experienced symptoms or preferences for care. Previous cross-sectional research has shown that cancer patients and survivors with depressive symptoms do experience not only more, but also *different* symptoms of depression compared to the general population [3, 8, 9]. Particularly, those living with and beyond cancer report more somatic symptoms, such as fatigue and sleep problems [8, 10]. Although it has been debated whether somatic symptoms such as fatigue should be included in the assessment of depressive symptoms in cancer survivors, recent research concluded that these somatic symptoms can help to discriminate different levels of depressive symptom severity and should therefore indeed be included [11].

Psychological treatments offered to cancer survivors for managing depressive symptoms, such as cognitive behavioral therapy, have a strong focus on cognitive-affective symptoms. These treatments might therefore not completely align with cancer survivors’ experienced symptoms or needs and could negatively influence care uptake and satisfaction with psychological care [12, 13]. Previous research already made clear that cancer patients and survivors want to receive help in managing the emotional impact of cancer, understanding their illness, and knowing their treatment options [14]. Research also examined *how* cancer patients and survivors prefer to receive care, i.e., they want it to be easily accessible and available, delivered by specialized psychologists, and integrated in cancer care [15]. However, it is not clear for *which symptoms* of depression cancer survivors would like to receive care. This is important to examine since it will allow psychosocial care providers to better align psychological interventions with survivors’ needs.

Recently, there has been a shift towards considering depression as a system. In this so-called network approach, depressive symptoms are considered to interact with and influence each other over time, rather than act as distinct indicators of a latent construct [16, 17]. For instance, fatigue might negatively influence the ability to concentrate on activities, which could in turn decrease the enjoyment of activities and make one feel more depressed. Previous cross-sectional network research found that depressive symptoms (both cognitive-affective and somatic) were less strongly interrelated in cancer patients and survivors than in the general population [8]. Fatigue, together with a depressed mood and loss of interest, was the most central symptom in cancer survivors, that is, most strongly related to other symptoms [8, 18]. Another cross-sectional network study in cancer patients and survivors found that a depressed mood and loss of enjoyment were the most central symptoms and thus most strongly related to other symptoms [19].

Although these previous studies have provided valuable insights into the interrelations of symptoms, the use of a cross-sectional between-subjects design did not allow the examination of whether and how certain symptoms *precede* other symptoms. A dynamic network study provides information about whether a symptom predicts other symptoms at a later time or is itself predicted by other symptoms at an earlier moment and is needed to learn more about the interplay of cancer survivors’ depressive symptoms over time [20–24]. Moreover, although still debated, it has been proposed that especially symptoms that predict other symptoms at a later time might be interesting intervention targets [22, 25]. One important aspect that has been overlooked so far is to what extent identified symptoms in a network approach align with cancer survivors’ personal experience of symptoms and their need for care. Greater insight into how these two align might add to the ongoing debate of how to use results from network analyses in clinical practice and treatment [22, 26].

Using longitudinal network data, the aim of this study is to gain a better understanding of cancer survivors’ care needs for distinct symptoms of depression; how depressive symptoms are interrelated in cancer survivors, both concurrently and over time; and whether survivors’ care needs correspond to the interconnectedness of specific depressive symptoms.

## Method

### Study design

This study used a cross-sectional survey study and an ecological momentary assessment (EMA) method. The study was reviewed and approved by the Medical Ethical Committee of the University Medical Center Groningen (METc 2017.600) and was performed in accordance with the ethical standards as laid down in the 1964 Declaration of Helsinki.

### Participants

We included cancer survivors who received a cancer diagnosis in the past 5 years and had finished their cancer treatment at least 3 months prior to study participation. They also had to experience at least mild levels of depressive symptoms (PHQ-9 ≥ 5), were 18 years or older, and able to complete questionnaires in Dutch. We excluded survivors who were already receiving psychological care.

Recruitment was done in cooperation with the Department of Radiation Oncology of the University Medical Center Groningen. This department compiled a list of all cancer survivors who received a cancer diagnosis in the past 5 years (excluding breast cancer survivors, as this group was already participating in other studies) and had finished their treatment over 3 months ago. Survivors were screened for depressive symptoms as part of standard care. Those who scored below the cutoff for mild depressive symptoms [27] received a letter from the Department of Radiation Oncology saying that they did not seem to experience depressive symptoms, but if they felt they needed psychological help they could contact their GP. Survivors with a sum score of five or higher on the PHQ-9 received a letter saying that they would be contacted by telephone to discuss their answers on the questionnaire. During the telephone call, participants were asked about aspects of their depressive symptoms, their cancer, and cancer treatment. At the end of the telephone call, those who met all inclusion and exclusion criteria were asked if they were interested in participating in a study about depressive symptoms. If they were, they received an information letter and informed consent form by post from the researchers.

### Procedure

Participants who submitted a signed informed consent form received the baseline questionnaire via e-mail. Once they had completed the baseline questionnaire, the researchers contacted them by telephone to discuss the preferred start date of the ecological momentary assessments (EMA), explain the EMA procedure, and sign them up to SurveySignal. SurveySignal is a mobile research platform that sends automated text messages with a link to a survey [28]. Participants received the EMA survey five times a day for 14 consecutive days on their smartphone. We used a semi-random sampling scheme that generated the assessment times randomly within fixed intervals [29]. Participants who did not own a smartphone with internet connection could borrow a smartphone from the University Medical Center Groningen. After filling in the diary, participants received a gift card and a personal feedback report including an overview of their given answers. Data were collected between October 2019 and October 2020.

### Measures

#### Demographic variables and cancer characteristics

Participants’ demographic variables (including age, gender, education, employment, and partner status) and cancer characteristics (including cancer type, cancer treatment, and time since diagnosis) were measured in the self-report baseline questionnaire.

#### Need for care for distinct depression symptoms

In the self-report baseline questionnaire, we measured participants’ need for care for distinct symptoms of depression, based on the Patient Health Questionnaire (PHQ-9). The PHQ-9 [27] is a validated and often used questionnaire to measure depressive symptoms in cancer survivors as defined by the DSM-V. For each of the nine PHQ-9 symptoms, we asked if participants experienced a need for care with the question: “In the past two weeks, I felt a need for care for…” which was supplemented with the corresponding item of the PHQ-9 [27] and could be answered with “yes,” “maybe,” or “no.” For instance, we measured need for care for little enjoyment with the item: “In the past two weeks, I felt a need for care for having little interest or pleasure in doing things.”

#### Dynamic network symptoms

In the EMA, we measured depressive symptoms with eight nodes that were measured with one item each (e.g., at the moment I feel down) to which participants had to indicate to what extent they agreed with the statement using a VAS scale ranging from zero (not at all) to 100 (to a large extent) (see Supplementary Appendix A for an overview of the EMA items). Five of the eight nodes (i.e., little enjoyment, feeling down, fatigue, feeling inadequate, and lack of concentration) were based on related PHQ-9 symptoms. The remaining four PHQ-9 symptoms could not be measured in the EMA: either because they would not show enough variability if they would be measured five times a day (i.e., for sleep problems, changes in appetite, and agitation or retardation) [29] or because we did not want to burden participants with this question five times a day (i.e., suicidality). In addition to the five nodes based on the PHQ-9, we added three nodes (i.e., anxiety, irritability, and worry) because these symptoms are prevalent in cancer patients and survivors and often cluster with depressive symptoms [30–32].

### Statistical analyses

We preregistered the data analysis plan on the online Open Science Framework (https://osf.io/6x3f4/). Statistical analyses were performed in R (version 1.4). We calculated means and standard deviations or counts and percentages for the demographic and clinical characteristics. We examined participants’ need for care for distinct depressive symptoms by calculating counts and percentages. Before examining the dynamic networks, we checked whether the included nodes varied sufficiently by calculating the mean squared successive difference (MSSD) [33]. This showed that all nodes indeed had sufficient variability (MSSD > 50) [34]. Additionally, Augmented Dickey-Fuller (ADF) tests showed that all nodes were stationary (*p*-value < 0.05) [35]. In other words, the means and variance of the data were not dependent on time [36].

We used the R package “mlVAR” to estimate two symptom networks: a contemporaneous network and a temporal network. This package removes all edges that are likely to be spurious (not significantly different (α = 0.05) from zero) [36] from the model to make it easier to interpret and was used to plot both networks visually. The contemporaneous network shows how symptoms predict each other at the same time point [37] and consists of partial contemporaneous correlations—the association between two nodes after controlling for all other nodes in the network [16]. We calculated the node strength to examine how strongly each node was connected to other nodes [23, 24]. Strength centrality is the most suitable and therefore most often used indicator of centrality in psychological networks [22].

Next, we plotted a temporal network which shows how symptoms are predicted by all symptoms at t-1, including autocorrelations [37]. The temporal network consists of partial directed correlations that entail how strong and in what direction two nodes are associated after controlling for all other nodes in the network [16]. We calculated the indegree—which shows how much information a node receives from other nodes in the network—and the outdegree of the nodes—which shows how much information a node sends to other nodes in the network [38].

## Results

### Participant recruitment, demographic, and cancer-related characteristics

In total, 1738 cancer survivors were approached as part of standard care, of which 1358 (78%) returned the screening questionnaire (see Supplementary Appendix B). Of the 70 survivors who gave informed consent, six resigned their participation, two did not have a smartphone to fill in the questionnaires,^1^ and one could not be reached. Of the remaining 61 included participants, eight did not fill in enough EMA measurements (< 60%, i.e., less than 42 out of 70 measurements) and one indicated in the baseline questionnaire to receive psychological care^2^ and thus did not fulfill our criteria. This resulted in a final sample of 52 participants who were included in the analyses, with a total of 3178 measurements (87% of all measurements were filled in).

The demographic and clinical characteristics of our sample of 52 cancer survivors are shown in Table 1. The majority of participants were male (67.9%) and on average 62 years old. The 52 participants who filled in the EMA were comparable to the group who did not participate in the EMA, apart from the fact that participants in the EMA were significantly younger (on average 62 years compared to 68 years in the non-participating group) and had significantly more hematological cancer (19% compared to 5% in the non-participating group). Table 1 also shows means and standard deviations of participants’ depressive symptoms at baseline. As can be seen, S*leep problems* and *Fatigue* were reported most frequently (EMA data confirmed that fatigue was highly reported, see Supplementary Appendix A).

**Table 1:** Demographic variables, cancer characteristics, and depressive symptoms at baseline (*N* = 52)

	*N* (%) or mean (*SD*)
Gender (female)	17 (32.1%)
Age (in years)	62 (15.0)
Education	
Low	16 (30.2%)
Middle	22 (41.5%)
High	15 (28.3%)
Employment	
Retired	20 (37.7%)
Paid job	14 (26.4%)
Inability to work	7 (13.2%)
Homemaker	5 (9.4%)
Other^†^	7 (13.2%)
Partner status (%)	
Married, registered partnership or living together	45 (84.9%)
Single	3 (5.7%)
Other^‡^	5 (9.4%)
Cancer type (multiple cancer types possible)	
Male reproductive organs	22 (41.5%)
Digestive system cancer	19 (35.8%)
Hematology	10 (18.9%)
Other^§^	17 (32.1%)
Cancer treatment (multiple treatments possible)	
Surgery	33 (62.3%)
Radiotherapy	53 (100%)
Chemotherapy	22 (41.5%)
Hormonal therapy	7 (13.2%)
Immunotherapy	2 (3.8%)
Other	1 (1.9%)
Time since (last) diagnosis (in years)	2.79 (1.03)
Total PHQ-9 score^¶^	7.98 (4.37)
Little enjoyment	0.96 (0.71)
Feeling down	0.75 (0.65)
Sleep problems	1.65 (1.06)
Fatigue	1.62 (0.89)
Changes in appetite	0.65 (0.95)
Feeling inadequate	0.62 (0.82)
Lack of concentration	0.98 (0.96)
Agitation/psychomotor retardation	0.50 (0.80)
Suicidality	0.25 (0.59)

^†^Including searching paid work, receiving education, and doing voluntary work. ‡Including widow/widower, divorced, and having a partner but not living together. §Including urinary tract, respiratory tract, skin, female reproductive organs, and sarcoma. ¶ The total PHQ-9 score ranged from 0 to 27, the scores for individual items from 0 to 3

### Need for care for distinct depressive symptoms

In total, 18 cancer survivors reported that they (maybe) wanted to receive psychological care and they were asked to what extent they wanted care for *distinct* symptoms. The four most often reported symptoms for which these participants wanted care were the following: *Fatigue, Sleep problems, Feeling down,* and *Little enjoyment* (see Table 2). Seven participants had missing data on the question about need for care. As they indicated earlier in the questionnaire that they already received care for their symptoms, they were not asked about a need for care. When analyzing the data, it became clear that all these participants received physical care for somatic symptoms such as fatigue, for instance, from a physiotherapist. The remaining 27 participants did not want to receive psychological care and were.

**Table 2:** Participants’ need for care for distinct symptoms (*N* = 18)

Symptom	Care need (yes or maybe) *N* (%)
Little enjoyment	13 (72.2%)
Feeling down	14 (77.8%)
Sleep problems	14 (77.8%)
Fatigue	17 (94.4%)
Changes in appetite	6 (33.3%)
Feeling inadequate	8 (44.4%)
Lack of concentration	9 (50.0%)
Agitation/psychomotor retardation	7 (38.9%)
Suicidality	4 (22.2%)

Participants’ need for care for distinct symptoms was only measured in participants who indicated to (maybe) have an overall need for care

### Contemporaneous network

**Fig. 1:**
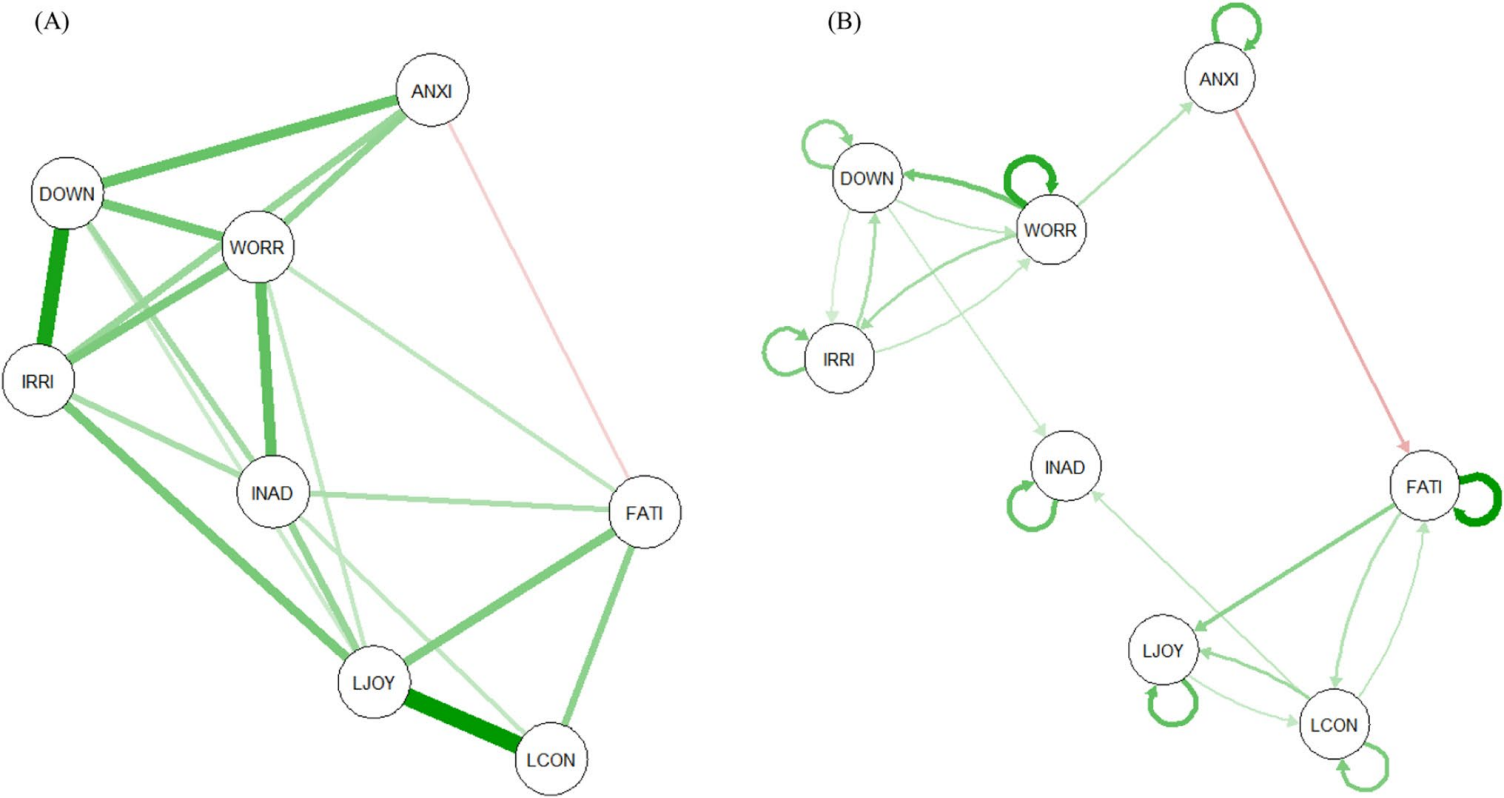
Contemporaneous (**A**) and temporal network (**B**) of depressive symptoms. Note*.* Thickness of the edges represents the strength of a connection between two nodes. Positive connections are shown with green edges, negative connections with red edges. LJOY = little enjoyment, DOWN = feeling down, FATI = fatigue, INAD = feeling inadequate, LCON = lack of concentration, ANXI = anxiety, IRRI = irritability, WORR = worry

**Fig. 2:**
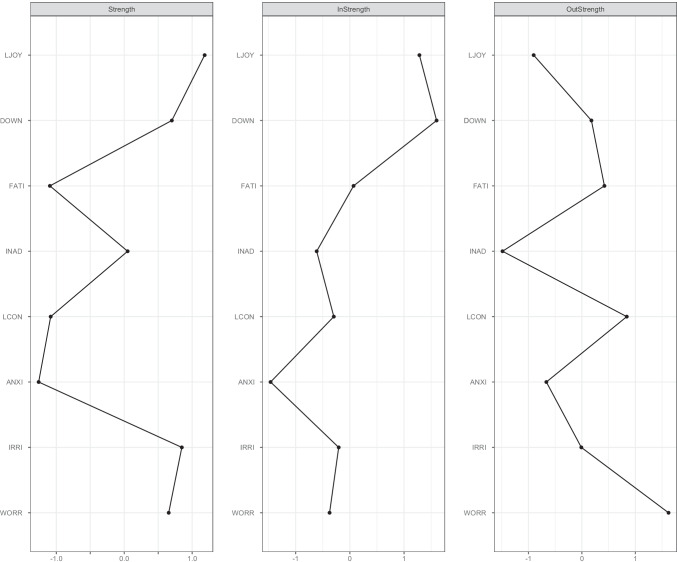
Centrality plots for the contemporaneous and temporal network. Note*.* Strength relates to the contemporaneous network; InStrength and OutStrength relate to the temporal network. LJOY = little enjoyment, DOWN = feeling down, FATI = fatigue, INAD = feeling inadequate, LCON = lack of concentration, ANXI = anxiety, IRRI = irritability, WORR = worry

The strongest connections in the contemporaneous network were between *Little enjoyment* and *Lack of concentration* and between *Irritability* and *Feeling down* (see Fig. 1A). This means that experiencing little enjoyment co-occurred with a lack of concentration, controlled for all other symptoms in the network. Similarly, high levels of irritability were related to high levels of feeling down and vice versa, taking into account other symptoms in the network. Three of these four symptoms (i.e., *Little enjoyment*, *Irritability*, and *Feeling down*) were also the most central in the contemporaneous network, together with *Worry* (see Fig. 2). This means that these symptoms were most strongly connected to other symptoms at the same point in time. Supplementary Appendix A shows the means and standard deviations for all nodes measured during the EMA.

### Temporal network

The temporal network showed the strongest edges for the autocorrelations of *Fatigue* and *Worry* (see Fig. 1B). This indicates that *Fatigue* and *Worry* were strongly predicted by their scores at t-1, controlled for all other nodes at t-1. *Feeling down* and *Little enjoyment* had the highest indegree, which shows that these symptoms were most strongly affected by other symptoms in the network one time point earlier (see Fig. 2). *Feeling down* was most affected by prior feelings of *Worry*, whereas *Little enjoyment* was most affected by preceding levels of *Fatigue*.

*Worry* had the highest outdegree of all nodes in the network, meaning that *Worry* sent the most information to subsequent symptoms in the network. As can be seen in Fig. 1B, more worrying was related to higher levels of *Feeling down*, *Irritability*, and *Anxiety* one time point later. *Fatigue* and *Lack of concentration* also had relatively high outdegrees. For both nodes, their strongest connection was with *Little enjoyment*. Higher levels of *Fatigue* and *Lack of concentration* were thus related to higher subsequent levels of *Little enjoyment*.

### Comparing cancer survivors’ preferences to their dynamic networks

When comparing participants’ need for care for distinct symptoms (i.e., *Fatigue, Sleep problems, Feeling down, Little enjoyment)* with the network results, it can be seen that *Feeling down* and *Little enjoyment* were also most central in the contemporaneous network (i.e., most strongly connected to other symptoms) and most strongly influenced by preceding symptoms in the temporal network. *Fatigue* was strongly predictive of other subsequent symptoms (mainly *Little enjoyment*) in the temporal network. *Sleep problems* were not measured in the EMA and we could therefore not examine its role in the network. *Lack of concentration* and *Worry* also had high outdegrees in the temporal network, but were not high (i.e., *Lack of concentration*) or not measured in participants’ need for care (i.e., *Worry*).

## Discussion

The current longitudinal study was the first to examine cancer survivors’ need for care for distinct depressive symptoms and how their care needs for specific symptoms related to their depressive symptom networks. Most participants liked to receive care for fatigue, feeling down, little enjoyment, and sleep problems. Feeling down and little enjoyment were also the most central symptoms in the contemporaneous network (i.e., most strongly connected to other symptoms) and were most strongly predicted by preceding symptoms in the temporal model (e.g., by worry and fatigue, respectively). Fatigue, the number one symptom participants wanted to receive care for, was most strongly related to subsequent symptoms, particularly little enjoyment. It should be noted that sleep problems, for which participants also reported a need for care, could not be examined in the network analyses.

A key finding was that feeling down and little enjoyment, for which cancer survivors reported a need for care, were the most central in the contemporaneous network of depressive symptoms. This is in line with these symptoms being considered the two core symptoms of depression, according to the DSM-V [39]. Previous cross-sectional network studies have also shown that depressed mood and lack of enjoyment were central in the network of depressive symptoms among cancer patients and survivors [8, 19]. This finding is also confirmed by two recent reviews including cross-sectional network studies among the general population [22, 40]. Over time, feeling down and little enjoyment were strongly predicted by the presence of other symptoms one time point earlier, particularly by fatigue and worry. This is consistent with previous research in the general population [41–43]. Many participants preferred to receive psychological care for feeling down and little enjoyment, possibly indicating a high experienced burden of these symptoms. Results suggest that current psychological treatments focusing on reducing cognitive-affective symptoms do align with survivors’ experienced affective symptoms as well as preferences for care. As such, there does not seem to be a mismatch, making it questionable whether this can explain cancer survivors’ low psychological care uptake.

Findings for fatigue were more mixed, which is in line with findings from previous studies. In the contemporaneous network, fatigue was not among the most central symptoms, which is similar to findings of a cross-sectional network study in cancer patients and survivors seeking psychological care [19]. In contrast, in another study, fatigue was central in the depression symptom network in a large sample of cancer patients and survivors as well as in the general population [8]. Two systematic reviews of cross-sectional network studies in the general population also found that fatigue (or loss of energy) was a central symptom in depression [22, 40]. One explanation for these inconsistent findings may be the differences in the sample (e.g., size or type of sample) and the assessment of fatigue (e.g., one or multiple item assessment, or the time scale which may range from everyday momentary states to retrospective reports of symptoms in the past week or weeks) [22, 25, 40].

In our temporal network, fatigue was one of the symptoms that most strongly predicted other symptoms at a later point in time, such as feeling down and little enjoyment. This is in line with previous network research in the general population [41]. This result extends previous cross-sectional findings that have shown fatigue to be a central node in contemporaneous networks [8, 18, 22]. It has been argued that fatigue can act as a bridge symptom between somatic illnesses such as cancer and depression [44, 45]. When cancer survivors are fatigued (for instance because of a long-term consequence of the cancer treatment), they may be more likely to develop psychological problems including symptoms of depression and anxiety [16, 44]. Fatigue was the number one symptom for which cancer survivors reported a need for care. Overall, results could indicate that intervening on fatigue might be beneficial for many cancer survivors as it could have an impact on other symptoms of depression. Psychological interventions targeting cancer-related fatigue, such as those based on mindfulness or cognitive behavioral therapy, have indeed been found to be effective in reducing fatigue in cancer survivors [46, 47].

Regarding worry, the few studies that examined the dynamic network of depressive symptoms did not include worry (or rumination). Our results suggest that worry may play an important role in the network of depressive symptoms, by predicting other symptoms at a later point in time. Therefore, reducing worry may be an important target for interventions in depressed cancer survivors. This result aligns with the assumption that worry is a key component or mechanism underlying a range of mental illnesses including depression and anxiety and may worsen and prolong the duration of a negative, depressed mood [39, 48, 49]. Psychological interventions including mindfulness-based and cognitive-behavioral interventions have proven to be effective in reducing depressive symptoms by reducing worry and rumination [50–52], also in people who have had cancer [53, 54].

### Clinical implications

It might be beneficial for clinical practice to pay specific attention to the presence of fatigue, lack of concentration, and worry when screening cancer survivors for psychological problems, as these symptoms were most predictive of the intensity of other symptoms at the next time point [22]. Furthermore, it can be highlighted by health care professionals, when providing psycho-education, that many survivors have a need for care relating to fatigue, sleep problems, feeling down, and little enjoyment, in order to normalize the experience of these problems. Also, cancer survivors can be motivated to particularly seek care when experiencing fatigue, a lack of concentration, and worry, as these symptoms may easily pave the way to other symptoms. Furthermore, they may benefit from psychological interventions that target fatigue, such as mindfulness-based and cognitive-behavioral interventions, also if they mainly experience one or both of the core affective symptoms (i.e., feeling down and little enjoyment). Cancer survivors can be explained that these symptoms are often caused by fatigue, and that an intervention aiming to reduce fatigue may therefore be effective in improving these symptoms as well.

### Strengths and limitations

A strength of the current study is that by examining how symptoms predict each other within-person and over time, we were able to extend existing research that has mostly focused on retrospective assessments of symptoms using a cross-sectional design [21, 29]. Hereby, we also took into account the role of worry, anxiety, and irritability in the network of depressive symptoms. Previous research has suggested that these additional symptoms may play a role in the network of depressive symptoms [30–32].

When interpreting our results, several limitations need to be taken into account. First, most participants in our study did not report a need for care—which is in line with previous research [55, 56]—and therefore only a small group of cancer survivors reported their care needs for distinct symptoms of depression. Future research in larger samples is needed to validate our findings. Second, as was also reported by other network studies on depressive symptoms, we could not include all depressive symptoms of the PHQ-9 (for instance, because they could not be measured multiple times a day), which complicated the comparison of the dynamic networks to survivors’ need for care. Furthermore, as we only assessed survivors’ care needs for the PHQ-9 symptoms, we had no information about their care needs for the additional symptoms (i.e., anxiety, irritability, and worry) that were included in the network models.

## Conclusions

Cancer survivors reported a need for care for specific symptoms of depression, including fatigue, feeling down, little enjoyment, and sleep problems. Results from the network models suggest that interventions specifically targeting fatigue and worry might be effective in reducing not only fatigue and worry, but also other symptoms of depression.

## Supplementary Information

Below is the link to the electronic supplementary material.

Supplementary file1 (DOCX 67 KB)

## Data Availability

The data that support the findings of this study are available from the corresponding author upon reasonable request.

## Footnotes

1 Due to COVID-19 restrictions, we were not able to visit participants in their homes to provide them with a smartphone and explain how they could use the device to fill in the EMA.

2 This participant had not indicated to receive psychological care in the screening call.
