# Supplementary material for: Looking at individual symptoms: the dynamic network structure of depressive symptoms in cancer survivors and their preferences for psychological care
Source: J Cancer Surviv. 2022 Aug 17;18(2):479–88. doi: 10.1007/s11764-022-01246-4 (PMC9382609; doi:10.1007/s11764-022-01246-4)
Supplement: Supplementary file 1 — Supplementary file1 (DOCX 67 KB) [file 11764_2022_1246_MOESM1_ESM.docx]

**Looking at individual symptoms: The dynamic network structure of depressive symptoms in cancer survivors and their preferences for psychological care**

**Journal of Cancer Survivorship**

E.A. Bickel^1^, M.P.J. Schellekens^2,3^, J.G. Smink^1^, V.E.M. Mul^4^, A.V. Ranchor^1^, J. Fleer^1^ & M.J. Schroevers^1^

Correspondence: E.A. Bickel, University of Groningen, University Medical Center Groningen, Department of Health Psychology, the Netherlands, e.a.bickel@umcg.nl

**Appendices**

Appendix A. *Overview EMA items (N=52)*

| Variables | Item | Adjustments | Mean (SD) |
| --- | --- | --- | --- |
| Little enjoyment | At the moment I enjoy activities | Reverse coded | 39.73 (23.14) |
| Feeling down | At the moment I feel down |  | 21.65 (22.06) |
| Fatigue | At the moment I feel tired |  | 47.99 (24.73) |
| Feeling inadequate | At the moment I feel inadequate |  | 26.46 (24.49) |
| Lack of concentration | At the moment I can concentrate | Reverse coded | 40.84 (23.36) |
| Anxiety | At the moment I feel anxious |  | 14.59 (18.00) |
| Irritability | At the moment I feel irritable |  | 21.59 (22.19) |
| Worry | At the moment I am worrying |  | 23.11 (23.68) |

*Note.* All items could be answered with a 100-point VAS scale ranging from ‘not at all’ to ‘to a large extent’


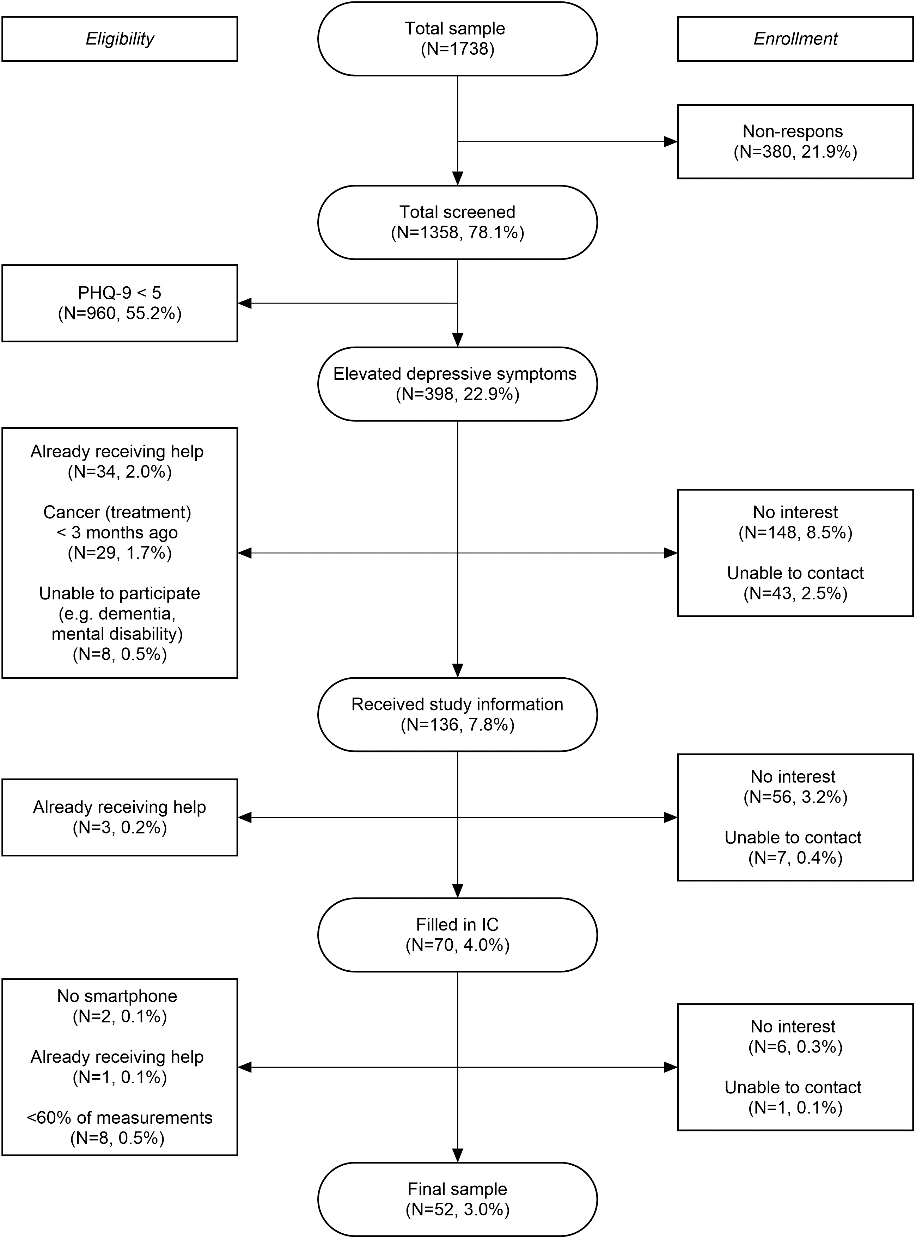


Appendix B. Participant recruitment and inclusion
